# Acute Effects of a Fungal Volatile Compound

**DOI:** 10.1289/ehp.8193

**Published:** 2005-08-09

**Authors:** Robert Wålinder, Lena Ernstgård, Gunnar Johanson, Dan Norbäck, Per Venge, Gunilla Wieslander

**Affiliations:** 1 Department of Medical Sciences/Occupational and Environmental Medicine, University Hospital, Uppsala, Sweden; 2 Division of Work Environment Toxicology, Institute of Environmental Medicine, Karolinska Institutet, Stockholm, Sweden; 3 Department of Medical Sciences/Clinical Chemistry and Asthma Research Center, University Hospital, Uppsala, Sweden

**Keywords:** 3-methylfuran, airway physiology, biomarker, building-related illness, fungi, hypersensitivity pneumonitis, lung, microbial volatile organic compound (MVOC), mold

## Abstract

Objective: 3-Methylfuran (3-MF) is a common fungal volatile product with active biologic properties, and previous studies have indicated a contribution to airway disease. The aim of the present study was to assess the acute health effects of this compound in humans.

Design: Acute effects were assessed via chamber exposure to (1 mg/m^3^) 3-MF.

Participants and measurements: Twenty-nine volunteers provided symptom reports, ocular electromyograms, measurement of eye tear film break-up time, vital staining of the eye, nasal lavage, acoustic rhinometry, transfer tests, and dynamic spirometry.

Results: No subjective ratings were significantly increased during exposure. Blinking frequency and the lavage biomarkers myeloperoxidase and lysozyme were significantly increased, and forced vital capacity was significantly decreased during exposure to 3-MF compared with air control.

Conclusions and relevance to clinical practice: Acute effects in the eyes, nose, and airways were detected and might be the result of the biologically active properties of 3-MF. Thus, 3-MF may contribute to building-related illness.

Controlled human exposure studies have shown acute dose–effect relations for exposure to volatile organic compounds (VOCs) with respect to odor and irritative symptoms (Mölhave et al. 1986, 1991). Also, histamine release from human bronchoalveolar cells has been shown after exposure to microbial VOCs (MVOCs) from indoor mold (Larsen et al. 1998). Up to 300 different compounds, including 3-methylfuran (3-MF), can be detected in indoor air (Berglund and Johansson 1996).

3-MF is formed by a broad spectrum of fungi (Börjesson et al. 1992) and can be used as a marker for the active growth of microorganisms in water-damaged buildings (Wessen and Schoeps 1996). The substance has a characteristic fungal smell. It is biologically active and binds covalently to tissue macromolecules after metabolic oxidation. In one study, increased indoor levels of 3-MF were significantly related to symptoms of airway obstruction (Smedje et al. 1996). Thus, 3-MF may be suspected to contribute to the exacerbation of pulmonary diseases (Boyd et al. 1978).

The aim of the present study was to assess the acute effects of 3-MF on the eyes, nose, and airways via a battery of physiologic and biochemical tests (Ernstgård et al. 2002). The choice of 3-MF was based on its chemical properties and previous epidemiologic associations with respiratory symptoms (Smedje et al. 1996).

## Materials and Methods

### Subjects and chamber exposures.

The study group consisted of 30 healthy volunteers (14 females) 20–54 years of age (mean ± SD, 33 ± 9 years) that were medically examined before the first exposure. Atopy was tested by laboratory verified IgE antibodies to common Swedish allergens: cat, dog, horse, birch pollen, timothy, mugwort, *Cladosporium herbarum*, *Dermatophagoides pteronyssinus*, and *Dermatophagoides farinae* (Phadiatop test; Pharmacia Diagnostics, Uppsala, Sweden); 43% of the volunteers had laboratory verified atopy. The volunteers were informed orally and in writing about the design of the study, possible hazards, and their freedom to discontinue participation at any time. The study was approved by the Regional Ethical Committee at the Karolinska Institute, Solna, Sweden, and written consent was obtained from the participants.

The subjects were exposed to clean air and 3-MF (1 mg/m^3^) in random order. Each exposure session lasted for 2 hr. Exposures were conducted during resting conditions with the subjects seated. Up to five subjects at a time were exposed. Exposures were performed from December through February to minimize possible interference with pollen exposure, with a minimum period of 2 weeks between the two exposure conditions. The exposures were carried out in a 20-m^3^ dynamic exposure chamber with 18–20 air changes per hour. The temperature and the humidity in the chamber were set to 24°C and 30%, respectively. Temperature and humidity were continuously recorded (Vaisala HMP 36, Vaisala, Helsinki, Finland) and logged (Squirrel Meter Logger 1200 Series, Grant Instruments, Cambridge, UK). 3-MF vapor was generated by injecting liquid solvent into inlet air by means of a high-pressure piston pump (Gilson 302, Gilson, Villiers-le-bel, France). The inlet air was dispersed throughout the entire chamber ceiling.

Air was sampled from the upper central part of the exposure chamber to monitor the concentration of the compound during exposures. The air samples were transferred through a Teflon-coated tube to a gas chromatograph by means of a pump (DDA-P101-BN, Gast, Benton Harbor, MI, USA). The gas chromatograph (Auto system; Perkin Elmer, Buckinghamshire, UK) was equipped with a wide-bore capillary column (CP-sil 8, 10 m, 0.53 mm inner diameter, 2 μm; Chrompack, Middleburg, the Netherlands) and a flame ionization detector. Helium was used as a carrier gas; the temperatures of the oven and the detector were 55°C and 250°C, respectively.

### Symptom questionnaire.

At six different times, subjects were asked to fill out a questionnaire with 10 questions related to smell, irritative symptoms (of the eyes, nose, and throat), dyspnea, headache, fatigue, dizziness, nausea, and intoxication. Answers were given by marking along a 100-mm visual analogue scale graded from “not at all” (0 mm) to “almost unbearable” (100 mm). The questionnaire was elaborated for vapor exposure and has been used in several inhalation studies (Ernstgård et al. 2002; Falk et al. 1991; Iregren et al. 1993; Nihlen et al. 1998).

### Blinking frequency.

Blinking of the left eye was recorded by electromyography (EMG) using three skin electrodes, two on the orbicularis oculi muscle and one reference electrode on the cheekbone. The EMG signal was amplified and transferred via telemetry to a personal computer. We used a software program in C++ to identify the characteristic EMG signal patterns. We identified blinks by comparison against nine conditions related to the size, shape, and appearance of the pattern (Ernstgård et al. 2002).

### Tear film break-up time.

Precorneal tear film stability was assessed by measuring the tear film break-up time by scanning the precorneal tear film with a biomicroscope (Topcon SL1E; Topcon, Tokyo, Japan). The time in seconds was recorded from the last blink until a rupture in the precorneal film was observed. We also estimated tear film stability by recording the self-reported tear film break-up time. The subjects were asked to keep their eyes open, and the time was recorded until they felt an urge to blink, assuming that this feeling was the appearance of a dry spot on the cornea (Wieslander et al. 1999). Measurements of break-up time were performed on three occasions in each eye: before entering the chamber, at the end of exposure, and 4 hr after exposure.

### Vital staining of the eye.

We assessed epithelial damage to the cornea and conjunctiva using a semiquantitative method. We instilled 4 μL of a dye, lissamine green (1% in physiologic saline solution), into the lower conjunctival sac. After 1 min, the cornea and conjunctiva were examined by a binocular microscope with a slit lamp (Topcon SL1E), and each eye was given a score of 0–9 (Norn 1991). Vital staining was performed once, 4 hr after exposure.

### Nasal lavage.

We measured inflammatory markers in nasal lavage samples before, directly after, and at 2 hr postexposure. Lavage of the nasal mucosa was collected with a 20-mL plastic syringe attached to a nose olive (Wålinder 1999). The analyses included myeloperoxidase (MPO), eosinophil cationic protein (ECP), lysozyme, and albumin and were carried out at the Department of Clinical Chemistry, University Hospital, Uppsala, Sweden. The chemical analysis of lavage biomarkers has been described in detail elsewhere (Wålinder 1999).

### Transfer test.

We determined the diffusion capacity of carbon monoxide (DLCO) using a single-breath technique (transfer test; PK Morgan Ltd., Chatham, Kent, UK) (Cotes et al. 1997; Forster et al. 1954). DLCO was measured for each subject before entering the exposure chamber and 20 min after leaving the exposure chamber.

### Dynamic spirometry.

Dynamic spirometric measurements were performed for each subject before entering the exposure chamber, immediately after leaving it, and 2 hr after leaving the chamber. Spirometric tests included vital capacity (VC), forced vital capacity (FVC), forced expiratory volume in 1 sec (FEV_1_), peak expiratory flow (PEF), and forced expiratory flow (FEF) in the middle half of FVC [FEF 25, 50, 75, the expiratory flows after one-fourth, one-half, and three-fourths, respectively, of the vital capacity has been expired (after a full inspiration)]. The tests were performed by spirometry (Vitalograph 2120 and Spirotrac 3 software for PC, version 2.0; Vitalograph, Buckingham, UK) according to the guidelines prescribed by the American Thoracic Society (1995).

### Acoustic rhinometry.

We assessed nasal patency using acoustic rhinometry. The nasal volume (from the nostril and 7 cm into the nasal cavity) and the minimal cross-section area were determined as an average of three measurements in each nostril. We performed the rhinometric measurements for each subject at three occasions during the exposure day: before entering the chamber, immediately after leaving it, and 2 hr after leaving it. Data on the nasal volumes and areas are presented as the sum of the right and the left side. The rhinometer, using a single-click signal of audible frequencies with the Nasal Area-Distance Acquisition Program, version 1.0 (University of Aarhus, Aarhus, Denmark), has previously been described by Hilberg et al. (1989).

### Statistical methods.

We compared the differences before and after exposure to 3-MF and air control using *t*-tests for paired samples for rhinometric and spirometric changes and Wilcoxon matched pairs tests for the non-normally distributed lavage data. We used repeated-measures analysis of variance (ANOVA) for subjective ratings and the blinking frequency series (Statistica for Windows, version 7.0; StatSoft Inc., Tulsa, OK, USA). Two-tailed tests and a 5% level of significance were applied when applicable.

## Results

### Suspected adverse reaction.

We removed one subject from the exposure series because of a two-phased pulmonary reaction to 3-MF. During the last 30 min of exposure, the subject suffered from moderate airway distress combined with acute airway obstruction. The PEF fell from 320 L/min before exposure to 170 L/min directly after. The acute dyspnea cleared up quickly, and by 3 hr after exposure, the PEF was 350 L/min. Three days after exposure, the subject had an onset of severe chest tightness, together with chills, fatigue, cough, and fever around 39°C. One week after exposure, the subject suffered from leukopenia, and obstructive symptoms remained for about a month. The subject’s chest X ray was normal, and no elevated titers for influenza virus A and B were found. Titers for total IgE and a mold-antigen panel were high. We reported the suspected adverse effect to the Regional Ethical Committee.

### Symptom ratings.

**Figure 1:**
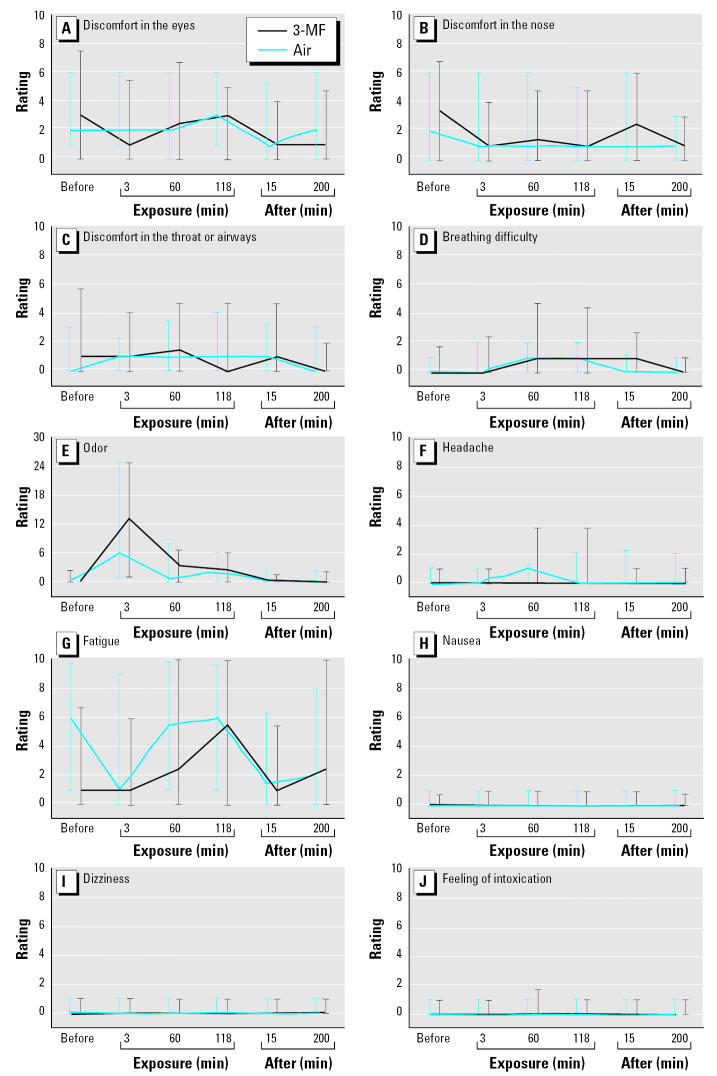
Subjective ratings (median with interquartile range, total range 0–100 mm) of 10 symptoms at six times: just before entering the chamber; at 3, 60, and 118 min of exposure; and at 15 and 200 min after exposure. Discomfort in the eyes (*A*), nose (*B*), and throat or airways (*C*); breathing difficulty (*D*); odor (*E*); headache (*F*); fatigue (*G*); nausea (*H*); dizziness (*I*); and feeling of intoxication (*J*).

The symptom ratings were not different during exposure to 3-MF compared with clean air (Figure 1). An immediate weak odor detection of 3-MF could be seen among some of the subjects, but not all (Figure E). This suggests that the exposure level was near the odor threshold and that adaptation occurred.

### Eye measurements.

**Figure 2:**
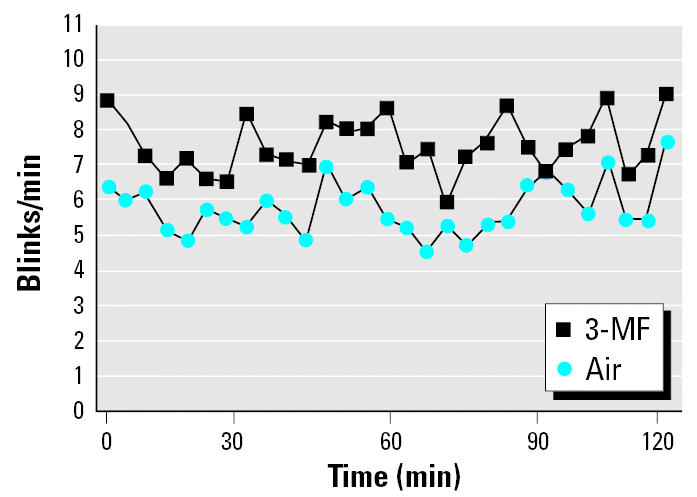
Blinking frequency during 2-hr exposures to 3-MF and air measured as mean frequencies every 2 min for 29 subjects.

The blinking frequency during 3-MF exposure was significantly higher than during clean air exposure (Figure 2, Table 1). The vital staining scores of epithelial eye damage detected with lissamine green were slightly higher after 3-MF exposure, but this effect was not statistically significant. The tear-film break-up time was significantly higher at the end of the 2-hr exposure period compared with the air control (Table 1). The observed changes were similar in subjects with or without atopy.

### Nasal measurements.

We observed a washout effect with decreased biomarker concentrations after repeated lavages following exposure to air. In contrast, compared with air controls, we observed an increase that was significant for MPO directly after and for lysozome 2 hr after exposure (Table 2). Nasal cavity dimensions, measured by acoustic rhinometry, were not different from air control (Table 3). Stratification by atopy did not show different reactivity for biomarkers or rhinometry, although baseline levels of ECP and albumin were doubled for subjects with atopy.

### Airway measurements.

On average FVC decreased 0.1 L directly after exposure to 3-MF, which was a significant decrease compared with air control. The other lung function parameters (transfer test, VC, FEV_1_, PEF) were not affected by exposure to 3-MF compared with clean air (Table 4). Stratification by atopy showed that the observed effect on FVC mainly appeared among nonatopics.

## Discussion

Although the exposure level of 3-MF was near the smell threshold and did not cause subjective symptoms of mucosal irritation or airway distress, the objective measurements did show effects on the eyes and airways. Considering an increase of the blinking frequency as an indicator of eye irritation together with the nasal biomarker response, it is possible that 3-MF might have mucosal effects in both the eyes and the airways. We also found an increased tear film break-up time after exposure to 3-MF. The tear film stability is dependent on the quality and amount of the fatty layer on its surface that is produced from the meibomian glands. The secretion from these glands is stimulated by the blinking movements, and a congruent increase of both blinking frequency and break-up time can be expected.

MPO is a marker of the neutrophil activity in the nasal mucosa, and lysozyme is a marker of both neutrophil activity and secretory neurogenic stimuli. Because nasal lavage was performed three times, a washout effect with decreased concentrations could be expected. We observed this decrease for all lavage bio-markers after exposure to air in contrast to an increase after exposure to 3-MF. Also, the decreased FVC after exposure to 3-MF indicates an airway effect. This pulmonary function variable is slightly more sensitive to airway irritation and hyperreactivity than is the VC measurement with slow expiration.

3-MF is metabolically activated via microsomal oxidation, cleaving the furan ring to a highly reactive unsaturated dialdehyde, methylbutenedial, that binds covalently to tissue macromolecules (Ravindranath et al. 1984). Animal inhalatory studies have revealed organ damage at high exposures. Haschek et al. (1984) reported that rats inhaling 1,000 mg/m^3^ 3-MF for 1 hr had damaged airway epithelium with pneumonitis and necrotizing suppurative rhinitis. They also observed necrosis, fibrosis, and epithelial metaplasia in the airways at autopsy 14 days later. Previous epidemiologic results also show airway reactions related to 3-MF in indoor air (Smedje et al. 1996).

This suspected adverse reaction was previously reported (Wålinder et al. 1998) in a subject with atopy who previously had been working in a mushroom farm and with micro-fungi. This subject suffered an acute obstructive reaction and a delayed pulmonary reaction with flulike symptoms. A nonspecific airway reaction could explain the immediate effects and an infection the late reaction, but no infection was verified by laboratory tests. Instead, analyses afterward showed mold allergy and high titers of IgE (Wålinder et al. 1998). Previous exposure to fungi at work could have resulted in a sensitization causing the present reaction to 3-MF. Hypersensitivity pneumonitis is an occupational disease from exposure to organic dust, fungi, or mold. One of the manifestations is called mushroom picker’s disease. The symptoms are similar to those of the present reaction but are mostly seen after exposure to high-molecular-weight organic chemicals. There are, however, low-molecular-weight chemicals that can cause immunologic responses, for example, isocyanates and acid anhydrides. It is possible that 3-MF after bioactivation is covalently binding to proteins of the mucosa, causing both chemical injury and a protein-hapten reaction resulting in airway inflammation and a hypersensitivity pneumonitis.

Short-term experimental studies differ in many aspects in relation to real indoor exposures. Indoor exposures involve a high number of substances, typically at concentrations 10–1,000 times lower than those used in experimental studies but with possible chemical interactions. Furthermore, domestic exposures are much longer. Therefore, it has been suggested that toxic effect estimates of indoor volatile compounds should be adjusted for long-term exposures compared with shorter exposures, at least for nonirritative effects (Damgård-Nielsen et al. 1997). Using this argument, it might be justifiable to apply higher concentrations of indoor agents in experimental chamber studies. Another important issue that must be considered is a difference in individual susceptibility. A “healthy volunteer bias” could underestimate the effects compared with persons who, because of long-term daily exposures, have acquired a form of sensitivity to “sick buildings.” Because persons with atopy are considered more sensitive to dampness, mold, or other disturbances of the indoor environment, subjects with IgE-mediated allergy to common allergens were recruited for the present study. However, results do not support the statement that persons with atopy report more symptoms or have a higher reactivity to this fungal metabolite. Actually, the only difference observed in reactivity was that nonatopics had a decrease in FVC after exposure to 3-MF, whereas no such effect was seen among the subjects with atopy.

In conclusion, we have recorded acute effects from the eyes, nose, and airways indicating mucosal reactive properties of 3-MF, which is commonly found in buildings affected by microbial growth. The mucosal effects could be induced by a possible chemical injury from the bioactivation of 3-MF. More unusual but severe effects, such as hypersensitivity reactions after exposure to fungi and molds, could also be explained by a protein-hapten reaction. Therefore, the results of the present study may have relevance for the judgment of health problems due to microbial emissions.

## Correction

The 3-min value for 3-MF in Figure 4E was incorrect in the original manuscript published online. The figure has been corrected here.

## Figures and Tables

**Table 1:** Eye measurements (mean ± SD) in 29 subjects exposed to 1 mg/m^3^ 3-MF or clean air for 2 hr.

	Measured break-up time (sec)			Self-reported break-up time (sec)				
Exposure	Before	After^a^	4 hr after^b^	Before	After^a^	4 hr after^b^	Blinking frequency during exposure^c^	Lissamine staining after exposure^d^
Air	36 ± 19	−3 ± 17	−3 ± 17	32 ± 19	3 ± 17	3 ± 14	5.8 ± 0.7	0.2 ± 0.3
3-MF	33 ± 21	6 ± 8*	−1 ± 16	35 ± 21	2 ± 20	−4 ± 19	7.6 ± 0.8**	0.3 ± 0.5

a End of exposure compared with before exposure; negative value indicates decrease.

b Four hours after exposure compared with before exposure; negative value indicates decrease.

c Blinking frequency (blinks per minute) during exposure.

d Epithelial damage score (0–9), measured by lissamine staining, 4 hr after exposure.

* *p* = 0.014 by Wilcoxon rank sum test.

** *p* < 0.001 by repeated-measures ANOVA.

**Table 2:** Nasal biomarkers (mean ± SD) in 29 subjects exposed to 1 mg/m^3^ 3-MF or clean air for 2 hr.

	Lysozyme (mg/L)			ECP (μg/L)			MPO (μg/L)			Albumin (mg/L)		
Exposure	Before	After^a^	2 hr after^b^	Before	After^a^	2 hr after^b^	Before	After^a^	2 hr after^b^	Before	After^a^	2 hr after^b^
Air	4.5 ± 2.3	−0.6 ± 1.9	0.2 ± 2.4	3.3 ± 4.7	−0.3 ± 3.4	−0.9 ± 3.0	42.2 ± 53.0	−10.2 ± 25.8	−14.2 ± 27.7	17.6 ± 22.4	−6.3 ± 15.2	−4.4 ± 18.0
3-MF	3.8 ± 1.9	0.3 ± 2.0	1.7 ± 3.0*	2.6 ± 4.5	0.4 ± 5.0	−0.3 ± 3.3	34.8 ± 44.2	4.8 ± 44.6*	−4.9 ± 27.7	14.1 ± 21.9	−0.4 ± 14.5	1.8 ± 12.1

a End of exposure compared with before exposure; negative value indicates decrease.

b Two hours after exposure compared with before exposure; negative value indicates decrease.

* *p* < 0.05 by Wilcoxon rank sum test.

**Table 3:** Nasal measurements (mean ± SD) in 29 subjects exposed to 1 mg/m^3^ 3-MF or clean air for 2 hr.

	Volume (cm^3^)			MCA (cm^2^)		
Exposure	Before	After^a^	2 hr after^b^	Before	After^a^	2 hr after^b^
Air	9.7 ± 1.7	−1.0 ± 0.8	−0.9 ± 1.2	0.9 ± 0.2	0.0 ± 0.1	0.0 ± 0.2
3-MF	10.0 ± 2.1	−0.8 ± 1.7	−0.8 ± 1.7	0.9 ± 0.2	−0.1 ± 0.1	0.0 ± 0.2

MCA, minimal cross-section area.

a End of exposure compared with before exposure; negative value indicates decrease.

b Two hours after exposure compared with before exposure; negative value indicates decrease.

**Table 4:** Pulmonary function (mean ± SD) in 29 subjects exposed to 1 mg/m^3^ 3-MF or clean air for 2 hr.

	FVC (L)			FEV_1_ (L)			PEF (L/min)			DLCO (μmol/sec/kPa)	
Exposure	Before	After^a^	2 hr after^b^	Before	After^a^	2 hr after^b^	Before	After^a^	2 hr after^b^	Before	After^c^
Air	4.8 ± 1.0	0.0 ± 0.2	−0.1 ± 0.2	4.0 ± 0.8	0.0 ± 0.2	−0.1 ± 0.2	510 ± 120	0 ± 30	−20 ± 40	220 ± 60	0 ± 30
3-MF	4.9 ± 1.0	−0.1 ± 0.2*	−0.2 ± 0.2	4.0 ± 0.8	−0.1 ± 0.2	−0.2 ± 0.2	500 ± 120	−10 ± 40	−20 ± 40	220 ± 60	10 ± 30

a End of exposure compared with before exposure; negative value indicates decrease.

b Two hours after exposure compared with before exposure; negative value indicates decrease.

c Twenty minutes after exposure compared with before exposure.

* *p* < 0.05 by *t*-test.
